# Areas Where the Process of Adult Certificate of Visual Impairment Registration Needs Improvement: A Systematic Review

**DOI:** 10.7759/cureus.100605

**Published:** 2026-01-02

**Authors:** Alexander J Sherlock, Sara Sarhadi, Annalise M Tanaka

**Affiliations:** 1 Ophthalmology/Oncology, Queen Elizabeth Hospital Birmingham, Birmingham, GBR; 2 Education, Queen Elizabeth Hospital Birmingham, Birmingham, GBR; 3 Hospital Medicine, Queen Elizabeth Hospital Birmingham, Birmingham, GBR

**Keywords:** certification, ophthalmology, optometry, patient experience, visual impairment

## Abstract

The Certificate of Vision Impairment (CVI) plays a crucial role in the United Kingdom (UK), connecting individuals who are sight-impaired or severely sight-impaired to much-needed social support services. However, there is an inconsistent application of the service, with many eligible adults not receiving certification or subsequent registration, limiting their access to vital assistance. This systematic review synthesizes current evidence to identify where the adult CVI pathway in the UK may be falling short and how it might be improved. These shortfalls will include factors affecting both the certification and registration stages of the process.

Following Preferred Reporting Items for Systematic Reviews and Meta-Analyses (PRISMA) 2020 guidelines, a systematic search of MEDLINE (Ovid) and PubMed was conducted to identify qualitative, quantitative, and mixed-methods studies relating to CVI certification and registration in UK adults. Studies were included if they addressed patient or professional experiences, barriers to certification, or administrative procedures. Risk of bias was assessed using the Mixed Methods Appraisal Tool (MMAT, version 2018). A mixed-methods convergent synthesis was used to integrate experiential evidence with measurable service outcomes, combining thematic synthesis of qualitative findings with a narrative summary of quantitative data to examine how individual, professional, and system-level factors interact to shape CVI certification and registration in practice.

Eight studies were included, comprising data from over 9453 participants (including 8954 patients and 499 professionals). Four recurrent themes were identified: (1) limited knowledge among professionals regarding CVI registration and its benefits, (2) uncertainty among ophthalmologists in determining eligibility, (3) administrative delays to registration, and (4) the role and value of Eye Care Liaison Officers (ECLOs) and optometrists. Whilst qualitative data contributed to all themes, qualitative data supported the theme of limited knowledge among professionals regarding CVI registration and its benefits the most. ECLOs were consistently shown to improve both efficiency and patient experience, yet their availability and training varied significantly. Optometrists demonstrated strong accuracy in assessing eligibility for certification, supporting recent policy developments in Wales that expand their role in the process. However, widespread knowledge gaps and structural fragmentation remain.

A key limitation is that most included studies were qualitative and regionally focused, limiting generalisability, while heterogeneity in study designs and outcomes precluded formal meta-analysis.

Despite its intended purpose as a gateway to support, the UK’s CVI pathway remains inconsistently applied, resulting in missed opportunities for timely intervention and support. Professional education, clearer guidance on eligibility, investment in administrative infrastructure, and formal recognition of ECLOs and optometrists within the certification process may help reduce variation and improve equity of access. A nationally coordinated approach is needed to ensure that certification leads not only to registration but also to meaningful, timely support for those affected by visual impairment.

## Introduction and background

Visual impairment affects an estimated two million people in the United Kingdom (UK) and represents a major cause of disability, loss of independence, and social exclusion [1]. The Certificate of Vision Impairment (CVI), introduced in England in September 2005 to replace the BD8 form, serves as a formal referral mechanism for individuals who are sight impaired (SI) or severely sight impaired (SSI) to their local social services department (LSSD), enabling access to appropriate support and benefits based on individual needs [2]. The CVI was last updated in September 2018 by the Department of Health and Social Care (DHSC) [3,4]. 

Sight loss and blindness impose considerable public health and economic burdens [5]. In the UK, certification and registration form a two-stage process that underpins both patient support and public health monitoring. Certification mainly takes place in Hospital Eye Services (HES), but also in Low Vision Services in Wales [6]. England CVI data is submitted to the CVI Office at Moorfields Eye Hospital [7], Wales and Northern Ireland CVI data are collected independently, and Scotland does not currently collect certification data [8]. After certification, individuals can choose to be registered at their local council level, with registration typically including fewer individuals than those certified.

While registration data help plan local services, certification data provide a more robust basis for surveillance, allowing analysis of causes and prevalence of sight loss, informing national policy, research investment, and resource allocation [5]. This highlights the importance of accurate certification and registration data. For example, if fewer cases of age-related macular degeneration (AMD) are certified, the condition’s true prevalence will be underestimated. Consequently, it may receive less priority in policy decisions and resource allocation compared with other causes of visual impairment. It also provides insight into the significant geographic disparities in the services and potential inequalities in healthcare provision [9]. In 2013, the direct cost of blindness was estimated at £3 billion, with indirect costs at £5.7 billion [5]. Enhancing the accuracy and completeness of certification could strengthen both individual support pathways and broader prevention strategies. 

The referral process begins when a healthcare professional identifies a patient likely to meet SI or SSI criteria. With the patient’s consent, a consultant ophthalmologist conducts a formal assessment, completing the CVI based on visual acuity (VA), visual fields (VF), and support needs. Eye Care Liaison Officers (ECLOs) often assist with this process, though their availability varies across services, despite national standards recommending that ECLOs be present in all HES clinics and trained in line with Royal National Institute of Blind People (RNIB) guidance [10].

A recent development, following a joint statement from National Health Service (NHS) Wales Shared Services Partnership and Optometry Wales (29 May 2025), now allows qualified low vision practitioners and optometrists to complete CVI certification for eligible patients within primary care settings, including in patients’ own homes. This reform, effective 11 June 2025, expands certification beyond dry age-related macular degeneration to include all irreversible eye conditions in adults, enabling faster access to support and reducing reliance on hospital-based ophthalmologists [6].

Once completed, the CVI should be sent to the LSSD within five working days. The LSSD then contacts the patient to explain the benefits of registration and offer a personalised needs assessment [2].

**Figure 1:**
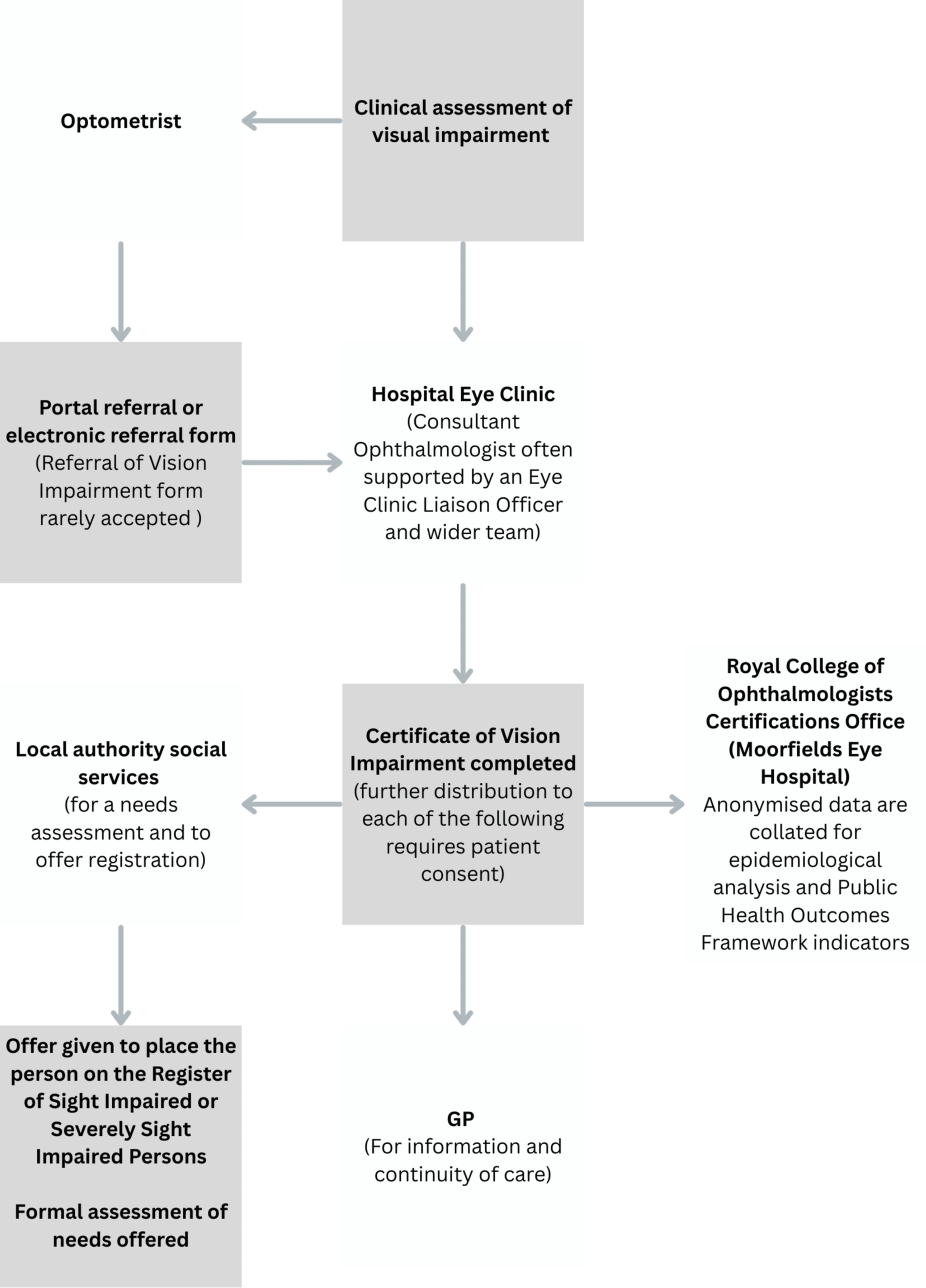
Schematic representation of the Certificate of Visual Impairment pathway in England. The flow diagram depicts the sequential pathway from initial referral to an ophthalmologist through to certification, registration, and subsequent access to associated social and health support services. The image is created by the author.

Figure 1 is a visual representation of the CVI registration pathway in England.

The CVI is essential in providing patients with SI or SSI with an appropriate level of care and allowing them to maintain confidence and independence, with many of the benefits shown in Appendix Table 6. 

Following successful certification and the patient’s informed consent, data may be used for epidemiological analysis contributing to NHS England’s Public Health Indicators reports [4,11]. The Public Health Outcomes Framework utilises certification data to monitor the burden of preventable sight loss across England [12]. CVI certification remains a principal source for estimating the causes of sight impairment and related demographic patterns, thereby supporting the evaluation of health inequalities [9]. However, the dataset is limited, as a substantial proportion of eligible patients are not offered certification. This is evident in the mismatch between disease prevalence and certification rates. For example, the prevalence of diabetic retinopathy was noted to have increased between 2009/10 and 2019/20; however, certification rates did not increase accordingly [12].

In August 2017, the CVI registration pathway in England was revised to address persistent issues reported by patients, clinicians, and social care services. The updates aimed to simplify the form, improve patient information, clarify certification guidance, and enhance data collection for service planning [7,12,13]. However, despite an increase of about 400,000 people aged 65 and over in the UK population [14], CVI registrations for AMD fell by 2.7%. This is despite both the size and the age of the population increasing, representing potential under-registration trends within the wider population [15]. The variations between disease prevalence and registration rates seem to be multifactorial. Literature identifies many different factors contributing to under-registration through diverse individual studies with limited systematic synthesis.

This systematic review synthesises qualitative, quantitative, and mixed-methods evidence on the adult CVI process in the UK. It aims to identify key barriers to timely and consistent certification and registration, examine facilitating factors, and explore the experiences of patients and professionals. The review also considers the impact of administrative processes and roles, including ECLOs and optometrists, on certification outcomes. By integrating diverse evidence, this review seeks to inform service improvement, professional training, and policy development to enhance the CVI pathway, patient experience, and improve equitable access to support.

## Review

Method

This systematic review employed a mixed-methods approach to synthesise evidence on the CVI certification and registration process for adults in the UK. The review adhered to Preferred Reporting Items for Systematic Reviews and Meta-Analyses (PRISMA) 2020 guidelines [16] and was prospectively registered in the International Prospective Register of Systematic Reviews (PROSPERO) (CRD420251234203- published on 18/11/2025), with minor amendments made on 09/12/2025 to note that forward citation searching was deemed unnecessary as it was considered unlikely to identify additional eligible empirical studies beyond those captured through database searching and backward citation checking [17].

Eligibility Criteria

Studies were eligible for inclusion if they examined any aspect of the CVI certification and registration process in adults (≥18 years) residing in the UK. 

Only English-language studies were included. All qualitative, quantitative, non-randomised, and mixed-methods study designs were eligible, including interviews, focus groups, cross-sectional studies, cohort studies, service evaluations, and database analyses. 

Eligible settings comprised hospital eye services, outpatient ophthalmology clinics, low vision clinics, optometry practices, ECLO services, and local authority departments responsible for visual impairment registration. The review was restricted to the UK context because the CVI system is specific to UK legislation and administrative structures.

Studies were included if they investigated any stage of the CVI pathway, including: assessment of eligibility for certification; provision and discussion of certification by ophthalmologists; completion and processing of CVI documentation; administrative procedures for submitting CVIs to local authorities; registration processes undertaken by social care services following certification; support provided by ECLOs or other allied professionals; involvement of optometrists in identifying eligibility or facilitating certification; referral pathways into the CVI process, such as from primary, community, or emergency eye care; factors contributing to under-certification, delay, or regional variability; patient or professional experiences of any of the above stages.

Eligible participants included patients with SI or SSI who were certified or registered (or were in the process of certification or registration) using the CVI pathway; healthcare professionals involved in certification or registration, including ophthalmologists, optometrists, nurses, and ECLOs; and social care professionals responsible for registration, assessment, or support of visually impaired adults.

Studies were excluded if they involved children or adolescents under 18 years of age; were conducted outside the UK; focused solely on general visual screening or epidemiology unrelated to CVI certification or registration; reported clinical interventions not linked to the CVI pathway; or were non-empirical publications such as editorials, commentaries, letters, theses, dissertations, or conference abstracts.

Information Sources and Search Strategy

Searches were conducted in MEDLINE (Ovid) and PubMed with no date restrictions, and the final search was completed on November 18, 2025, immediately following publication of the PROSPERO protocol. The search strategy combined controlled vocabulary and free-text terms relating to visual impairment, certification, registration, and service pathways. Full search strings are in the Appendix. Additional studies were identified through backward citation searching of all included articles. Grey literature was not systematically searched.

Study Selection

Titles and abstracts were screened independently by two reviewers using predefined eligibility criteria. Full-text articles were then assessed for inclusion. Discrepancies were resolved through discussion. A third reviewer was available if consensus was not reached.

Data Extraction

Data was extracted independently by two reviewers using a standardised form structured to reflect the summary table used in the review. The form captured key study characteristics, including design, setting, sample size, participant groups, data collection methods, and principal findings relevant to the CVI certification and registration process. It also included fields for identifying reported barriers, facilitators, and themes relating to awareness, clinical practice, and administrative processes. Extraction was conducted iteratively, with reviewers cross-checking entries for completeness and accuracy. Discrepancies in extracted data were discussed and resolved by consensus. A third reviewer was available if consensus was not reached.

Risk of Bias Assessment

Risk of bias was assessed using the Mixed Methods Appraisal Tool (MMAT) (version 2018) [18] by two independent reviewers. The MMAT was selected for its ability to evaluate qualitative, quantitative, and mixed-methods studies within a single framework, ensuring consistent appraisal across designs. It assesses key domains such as study design, data collection, analysis, and interpretation. Discrepancies were resolved by consensus. A third reviewer was available if consensus was not reached.

Data Synthesis

A mixed-methods convergent synthesis approach was used to integrate qualitative and quantitative findings. Qualitative data were analysed using thematic synthesis, employing an iterative coding process in which initial descriptive codes were generated from each study’s findings and refined into broader analytical themes through repeated cross-study comparison. Study characteristics and emergent themes were summarised in a comparative table to facilitate mapping of barriers, facilitators, and stakeholder perspectives. Quantitative data were narratively summarised due to heterogeneity in study design, participant populations, and outcome measures. Findings from both data streams were then integrated through convergent synthesis, aligning thematic patterns with quantitative indicators such as inter-rater agreement, certification and registration rates, and process timelines. Key themes were refined collaboratively and verified by reviewers. As the review was primarily qualitative and narrative, meta-analysis was not possible, and formal heterogeneity or sensitivity analyses were therefore not undertaken. Risk of bias assessments informed interpretation during data synthesis, with findings from studies at higher risk of bias interpreted more cautiously.

Results

**Figure 2:**
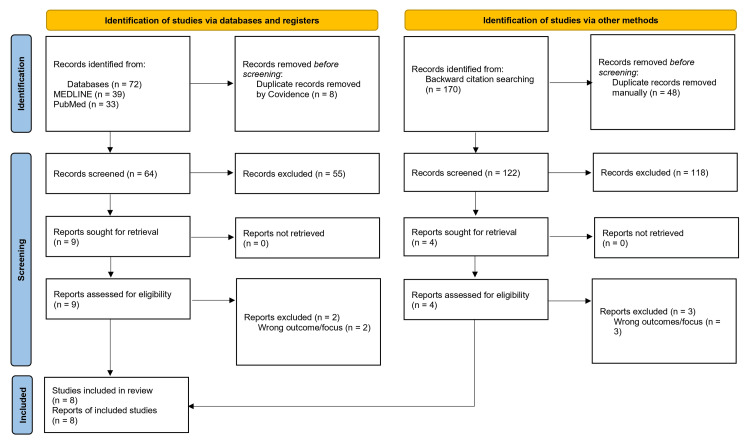
PRISMA flow diagram showing the identification, screening, eligibility, and inclusion process for studies included in the systematic review.

A total of 242 records were identified, which comprised 72 from electronic databases (MEDLINE, n = 39; PubMed, n = 33) and 170 from citation screening of relevant reference lists. Fifty-six duplicates were removed (48 manually and 8 via Covidence), after which 186 unique titles and abstracts were screened. Of these studies, 173 were excluded due to the predetermined exclusion criteria. Thirteen full-text articles were retrieved and assessed for their eligibility. Following the full-text review, five studies were excluded for examining outcomes outside the review scope. These included analyses of geographical variation or temporal trends in certification rates, descriptions of causes of sight impairment or specific disease burdens, and earlier studies assessing under-registration within pre-CVI systems (BD8). None investigated the procedural, administrative, or experiential aspects of the contemporary CVI certification and registration pathway. This process of identification and inclusion is denoted in Figure 2. The remaining eight studies met all inclusion criteria and were included in the final synthesis.

Analysis of Risk of Bias

Eight studies met the inclusion criteria and were appraised using the MMAT, as seen in Tables 1-3. Five qualitative studies met all MMAT criteria (Boyce et al. [2], Douglas et al. [19], Llewellyn et al. [20], Pardhan et al. [21], and Charlesworth et al. [22]), with coherence reported between data collection, analysis, and interpretation. The two quantitative non-randomised studies met most MMAT criteria. Bartlett et al. [23] involved voluntary participation, with a participating sample including only Welsh consultant ophthalmologists and low vision optometrists (LVOs). Findings may reflect the views of Welsh participants and may not reflect those of the wider UK population. Olvera-Barrios et al. [24] satisfied all MMAT criteria. Guerin et al. [25] was the only quantitative descriptive study. It invited 30 consultant ophthalmologists with experience in certification to rate 35 cases; participation was voluntary. As the sample consisted of consultants with certification experience and was voluntary, it may not reflect the broader population of UK ophthalmologists. The study did not report the number of non-respondents [25].

**Table 1:** Summary of methodological quality assessment of the included studies using the Mixed Methods Appraisal Tool for qualitative studies.

Study		Boyce et al. [2]	Douglas et al. [19]	Llewellyn et al. [20]	Pardhan et al. [21]	Charlesworth et al. [22]
Study design		Qualitative	Qualitative	Qualitative	Qualitative	Qualitative
Screening questions	Are there clear research questions?	Yes	Yes	Yes	Yes	Yes
	Do the collected data allow to address the research questions?	Yes	Yes	Yes	Yes	Yes
Qualitative	Is the qualitative approach appropriate to answer the research question?	Yes	Yes	Yes	Yes	Yes
	Are the qualitative data collection methods adequate to address the research question?	Yes	Yes	Yes	Yes	Yes
	Are the findings adequately derived from the data?	Yes	Yes	Yes	Yes	Yes
	Is the interpretation of results sufficiently substantiated by data?	Yes	Yes	Yes	Yes	Yes
	Is there coherence between qualitative data sources, collection, analysis and interpretation?	Yes	Yes	Yes	Yes	Yes

**Table 2:** Summary of methodological quality assessment of the included studies using the Mixed Methods Appraisal Tool for quantitative non-randomised studies.

Study		Bartlett et al. [23]	Olvera-Barrios et al. [24]
Study design		Quantitative non-randomised	Quantitative non-randomised
Screening questions	Are there clear research questions?	Yes	Yes
	Do the collected data allow to address the research questions?	Yes	Yes
Qualitative	Are the participants representative of the target population?	No	Yes
	Are measurements appropriate regarding both the outcome and intervention (or exposure)?	Yes	Yes
	Are there complete outcome data?	Yes	Yes
	Are the confounders accounted for in the design and analysis?	Yes	Yes
	During the study period, is the intervention administered (or exposure occurred) as intended?	Yes	Yes

**Table 3:** Summary of methodological quality assessment of the included studies using the Mixed Methods Appraisal Tool for quantitative descriptive studies.

Study		Guerin et al. [25]
Study design		Quantitative non-randomised
Screening questions	Are there clear research questions?	Yes
	Do the collected data allow to address the research questions?	Yes
Qualitative	Is the sampling strategy relevant to address the research question?	No
	Is the sample representative of the target population?	No
	Are the measurements appropriate?	Yes
	Is the risk of nonresponse bias low?	No
	Is the statistical analysis appropriate to answer the research question?	Yes

Summary of Included Studies

The characteristics, methodologies, and principal findings of the eight studies included in this review are summarised in Tables 4, 5. Table 4 outlines study design, participant groups, and data collection methods, while Table 5 presents key results and thematic interpretations relating to the CVI certification and registration process in the UK.

**Table 4:** Summary of studies included in the review examining the CVI registration process in the United Kingdom. This table presents key characteristics, methodologies, and principal findings of the eight included studies that explored various stages of the CVI pathway.

Study Title	Study aim	Study methodology		
		Study design	Participants	Data collection method
Boyce et al. [2]	To find out the experience of the patient, health care professionals and social care professionals in the certification and registration process. To also examine the main barriers to timely certification of patients.	Qualitative	43 health and social care professionals (ophthalmologists, nurses, optometrists, eye clinic liaison officers (ECLO), rehabilitation officers, administrators) and 46 patients certified as severely sight impaired (SSI) or sight impaired (SI) within the previous 12 months.	Semi-structured telephone interviews conducted across three National Health Service (NHS) hospital sites in England, selected for their differing certification of vision impairment (CVI) certification rates. Interviews were audio-recorded, transcribed, and thematically coded using inductive and deductive approaches.
Douglas et al. [19]	To find out the experience of patients going through vision impairment registration and opinions of the support after registration.	Qualitative	884 adults registered as severely sight impaired SSI or SI, across England, Scotland, and Wales (weighted to national register demographics). Subsamples: 395 participants registered in the previous 8 years (for certification-related questions) and 838 for service experiences in the prior year.	Structured face-to-face and telephone interviews. Standardised questionnaire covering experiences in eye clinics, certification, post-registration services, and family support. Data analysed descriptively and thematically from open responses.
Llewellyn et al. [20]	To explore the impact of ECLOs on patient care pathways, clinic efficiency, and their professional role.	Qualitative	141 health and social-care professionals across 30 ophthalmology clinics in England, Wales, Scotland, and Northern Ireland, including 26 ECLOs, 28 nurses, 24 consultants, 13 managers, 12 optometrists, 9 commissioners, 8 rehabilitation officers, and others.	Semi-structured interviews in person and over the phone. Conducted at 30 clinics with varying levels of ECLO provision. Interviews recorded, transcribed verbatim, and coded using NVivo 11 with a framework analysis approach.
Bartlett et al. [23]	To find the proportion of ophthalmologists and optometrists who agree on certification. To see if agreement was affected by bilateral atrophic age-related macular degeneration	Quantitative non- randomised	129 clinicians total. 30 consultant ophthalmologists and 99 low vision service Wales (LVSW) accredited optometrists. The consensus panel comprised 4 consultant ophthalmologists and 3 low-vision–accredited optometrists.	Each clinician independently rated 40 anonymised adult low-vision case records (randomly selected from 8,000 LVSW patients). Cases included varying severities of visual impairment and causes. Each case included visual acuity (VA), visual fields (VF), contrast sensitivity, and functional information. Raters classified each case as not eligible, SI or SSI via an online platform. Ratings were compared to the consensus panel’s decisions using logistic mixed-effects models and Krippendorff’s alpha for inter-rater reliability.
Pardhan et al. [21]	To identify current limitations to certification and explore these from the perspective of patients, optometrists and ECLOs.	Qualitative	25 participants total: 17 patients with various eye conditions, 4 ECLOs, and 4 community/primary-care optometrists	Semi-structured individual interviews. Conducted by a single interviewer to ensure consistency. Transcripts were thematically analysed using NVivo (v12) and synthesised via contextualist narrative analysis.
Charlesworth et al. [22]	To explore primary-care optometrists’ knowledge of the CVI and registration processes, to identify barriers and facilitators to supporting patients with low vision, and to assess attitudes toward optometrists certifying patients in the future.	Qualitative	148 optometrists in England (mean experience 21 years, 36 % male).	Online open-ended questionnaire
Guerin et al. [25]	To evaluate the level of ophthalmologist agreement when determining eligibility for visual impairment registration in patients with glaucoma and predominantly visual field loss.	Quantitative descriptive	30 consultant ophthalmologists across United Kingdom (UK) NHS hospitals	Each consultant rated 34 anonymised data sets containing VA (6/12 or better) and Humphrey 24-2 VFs from patients with glaucoma. Each rater independently categorised each dataset as SSI, SI, or neither. 16 consultants repeated the process after 1-3 months to assess intra-rater reliability.
Olvera-Barrios et al. [24]	To determine the prevalence of visual impairment in patients with diabetic retinopathy (DR), compare it with rates of formal CVI, and analyse demographic and clinical factors associated with visual impairment	Quantitative non- randomised	8 007 patients with DR referred from the English Diabetic Eye Screening Programme between 2016 and 2019	Extraction of clinical and sociodemographic data (visual acuity, DR grade, diabetes type, age, sex, ethnicity, index of multiple deprivation) from the hospital electronic records and the CVI audit database

**Table 5:** Summary of themes and findings identified from studies included in the review. AMD: age-related macular degeneration, VA: visual acuity, ECLO: Eye Clinic Liaison Officers, CVI: Certificate of Vision Impairment, NHS: National Health Service, RVI: referral of visual impairment, LVL: low-vision leaflet.

Study Title	Results and themes	Key findings/Interpretation	
			Boyce et al. [2]	Knowledge and awareness gaps: Most health professionals confused both certification and registration, assuming registration occurred as an automatic process after certification. Amongst health professionals there was limited awareness about social services’ role or the benefits of registration for patients.	Certification and registration processes are inconsistently applied. There were often delays due to confusion on when to refer a patient, either due to patient fluctuating condition or Ophthalmologists waiting for a disease course to finish. Administrative inefficiency and limited ophthalmologist knowledge led to frequent form errors and increased delays. ECLOs improve process efficiency and patient experience but are not consistently used across NHS Trusts. Greater education for professionals, patients and administrative staff could benefit the CVI process. More consistent use of ECLOs would likely improve the service.		
Uncertainty in clinical decision-making: Ophthalmologists had difficulty knowing when the right time was to certify a patient. This was particularly true for patients with conditions with a fluctuating course such as AMD. Many relied just on VA, disregarding functional vision or psychosocial needs.					
Certification is viewed as the “final stage” of treatment: Around half of the ophthalmologists delayed certification until medical treatment was exhausted, whereas patients saw certification as the beginning of receiving vital support.					
Administrative barriers and delays: There was considerable variability in how long certification and registration took (from weeks to nearly a year). Delays were often linked to incomplete forms, sending off forms as batches rather than promptly, and limited administrative support.					
Role of ECLOs: ECLOs had a significant role in expediting CVI completion, ensuring forms were correctly filled, and supporting patient understanding of the CVI process and available support. Hospitals with ECLOs reported fewer incomplete forms, noting prior to ECLOs up to 10-15% of forms would be returned. Both patients and clinicians praised ECLOs for improving communication and access to CVI related services.					
Patient impact: Patients described certification as emotionally overwhelming and ultimately life-changing. It increased their confidence and independence through social service interventions (mobility training, financial support, aids) leading to improvements in their quality of life.					
Douglas et al. [19]	Limited explanation at certification: 45% of participants reported receiving no explanation of registration at time of certification, 17% received only medical advice, 32% of working-age respondents received no help or information in the clinic.	Limited explanation during certification left many patients unclear about the process, benefits, or available support. Post-registration support was inconsistent and often delayed, with working-age adults reporting lower satisfaction. Many registered patients still felt deprived of needed practical and emotional support, highlighting the gap between certification, registration, and access to effective support and rehabilitation services Clear points of contact and integrated support improved satisfaction and service navigation, while variation across local authorities revealed systemic inequities and underscored the need for consistent follow-up to prevent patients being left unsupported.		
	Emotional and informational gaps: Shock, confusion, and lack of staged information delivery were reported frequently by patients. Many participants did not know what to expect or what services were available after registration.			
	Social/welfare service delays: 36% of patients were contacted by social/welfare services within 6 weeks of registration; 20% never received a visit. Strong regional variation was evident with the best authority having 88% of patients contacted in under 6 weeks, and the worst having 14% of patients contacted.			
	Limited support after registration: In the year after registration 17% of patients received no services and 53% of long-term registrants (registered for over 1 year) reported receiving no services at all in the previous year.			
	Differences by age and gender: Working-age adults compared to retirees were less likely to receive timely visits and were less satisfied with services (59% satisfaction amongst working age adults compared to 77% among retirees). Men reported higher satisfaction (87% satisfaction for men compared to 68% for women).			
	Themes from patient responses: Emotional impact: Shock and confusion at certification. Expectations: Low or negative expectations of support. Contact and continuity: The reassurance of having a point of contact was a particular source of satisfaction amongst many especially those over 65. Service fragmentation: There was confusion about boundaries between statutory and voluntary services amongst patients. Family support gaps: 87% of families of those involved in the service reported receiving no formal support.			
Llewellyn et al. [20]	Reducing administrative delays: ECLOs standardised and helped expedite CVI paperwork, reducing backlogs (“piles of CVIs waiting in consultant’s offices” no longer occurred)[20, p.3]. CVIs were sent to social services within 5 days in over 90 % of cases with ECLO involvement	ECLOs significantly improved clinic efficiency, patient experience, and coordination across health and social care, ensuring patients received necessary support and emotional guidance. Their integration into a service reduced administrative delays and clinician workload. In a discussion point by the author it was noted only 2 of 30 sites had fully integrated ECLOs within the formal NHS pathway. The authors recommended formalising the ECLO role in all eye clinics and quantifying cost savings and patient benefits in future research.		
	Improve non-clinical workload for clinicians: ECLOs relieved consultants and nurses of non-clinical duties, saving significant clinic time and allowing medical staff to focus on treatment and throughput (“releases time for trained staff to do what they trained for”) [20, p.4]			
	Continuity and holistic care: ECLOs acted to bridge the gap between clinical, social-care, and voluntary-sector services. This ensured suitable follow-up and provided patients with emotional and practical support. Their presence enabled smoother transitions from clinical care to ongoing rehabilitation and social support			
	The value of an ECLO is evident to surrounding staff: Participants agreed ECLOs improved patient experience and clinic workflow, staff in ECLO absent sites expressed that they wish to establish the role.			
	Simply appointing an ECLO is not enough: For an ECLO to positively impact the team it requires communication with the clinical team, managerial support, and suitable training and knowledge of local services.			
Bartlett et al. [23]	Accurate identification of patients requiring registration by Optometrists: The Optometrist group and consensus panel (4 consultant ophthalmologists and 3 optometrists with a formal accreditation in low vision) groups showed comparable agreement- median (interquartile range) number of cases in agreement = 33.0 (31.0-33.0) for ophthalmologists and 36.0 (34.0-36.5) for optometrists. For SSI cases: 76.0% (95% CI 71.4-80.1) for ophthalmologists, 61.8% (59.0-64.6) for optometrists. For SI cases: 51.6% (46.7-56.4) for ophthalmologists, 72.2% (69.8-74.5) for optometrists. For non-eligible cases: 94.2% for ophthalmologists, 87.8% for optometrists.	Low-vision optometrists showed equivalent accuracy to ophthalmologists in certifying sight impairment when compared with a consensus standard. It was shown that agreement was highest for cases of bilateral atrophic AMD. Findings suggest certification could occur in primary care, supporting expanded optometrist authority to enhance access and reduce delays. The authors recommend policy change to allow trained optometrists to complete CVIs for stable, untreatable conditions such as atrophic AMD.		
	Inter-rater reliability: There was moderate to substantial inter-rater reliability within each ratergroup consistency for ophthalmologists: α = 0.72-0.80 and optometrists: α = 0.67-0.73.			
	Observed differences between Optometrists and Ophthalmologists: Optometrists tended to make judgements based strictly on guidelines and more conservative judgements in borderline cases, ophthalmologists displayed more variability, possibly influenced by experiential and subjective judgment.			
Pardhan et al. [21]	Lack of clarity in the certification and registration process: Patients expressed confusion about the process, whether they were eligible and who would be responsible for initiating the registration process. Due to poor communication, many patients were not informed that certification does not automatically result in registration and this lead to feelings of frustration (“too bad to drive, not bad enough to be registered-I’ve fallen through a crack”) [21, p.3413]. ECLOs showed variability in their role as part of the CVI pathway, with some proactively referring patients to sensory services even without a CVI, while others awaited a decision from an ophthalmologist. Optometrists reported uncertainty about their role, rarely thinking to initiate the process themselves and often deferring to secondary care. (“we don’t really trigger or get involved much”)[21, p.3414].	The study identifies a major knowledge gap about the CVI process and the distinction between certification and registration, leading to uncertainty about eligibility, poor communication between professionals and patients, and unclear professional roles. Many patients “fell through the cracks” [p.3413] due to the absence of a standardised pathway. Poor coordination between secondary care and community services, along with limited additional role (e.g., optometrist) involvement, further contributed to under-registration. The authors recommend: (1) improved communication between clinicians, ECLOs, and social services; (2) standardised CVI/RVI pathways; (3) timely referral to third-sector support despite certification; and (4) national training to improve understanding of CVI, RVI, and LVL to reduce under-registration.		
	Delays in accessing certification and post-registration support: Patients were noted to have experienced significant and variable waiting times (from months to over a year) before social care contacting them. ECLOs reported local authorities often would require proof of certification before providing social services which meant delays in certification often had a knock-on effect (“the CVI is the gateway to services”)[21, p.3414]. Optometrists noted unclear referral routes and regional disparities contributed to process delays.			
	Confusion about support entitlements and access: Patients expressed mixed awareness of the practical or financial benefits of registration, with some believing “there are no benefits socially for being certified.”[21, p.3414]. Variability existed across local authorities in the services provided. ECLOs highlighted communication between hospital and community services was poor and patients were followed up inconsistently. Some patients were noted to be grateful for individual ECLOs but many reported to feel abandoned after diagnosis.			
	Professional variation and training gaps: ECLOs and optometrists both expressed insufficient understanding of CVI but also the RVI and LVL. They noted this lack of knowledge contributes to unequal patient experiences. Optometrists suggested improved training and awareness could help to resolve this issue (“there needs to be so much better training for us”) [21, p.3415].			
	Structural fragmentation: Not all NHS trusts have ECLOs and in addition, ECLO’s roles and funding in the CVI registration process appear to vary from one trust to another. This in turn, led to variable patient support. Some ECLOs were noted to not have the space or privacy to counsel patients.			
Charlesworth et al. [22]	Barriers to registration: This paper identified several barriers contributing to under-registration CVIs that stemmed from Optometrists. These barriers included limited knowledge of differences between CVI and RVI, uncertainty among professionals about referral pathways, low confidence, difficulty discussing sight loss given the emotional burden of the matter, and confusion about professional roles involved in the CVI registration process.	In presence of adequate training and funding, optometrists expressed they would be willing to be involved in the certification process. Extensive knowledge gaps were identified amongst professionals around CVI/RVI and local referral mechanisms. The study suggests the presence of a low-vision pathway which is funded nationally, can aim to reduce under-registration and waiting times. It can additionally provide support earlier to patients and improve communication within different professionals involved in the process.		
	System barriers: Logistics and system barriers often contributed to delayed registration. Some of these barriers are noted to be secondary to greater systematic factors such as short appointment times, limited funding of the low-vision pathway and long waiting times when it comes to receiving support. Some other factors included geographical location playing a role in accessing social services and low-vision aids with some patients having limited access to these.			
	Patient barriers: Some of the factors contributing to delayed CVI registration were related to patients themselves. Accepting sight impairment can be emotionally challenging and many were reluctant to accept loss of independence. Additionally despite available leaflets that can be shared with the patients, many are not well suited to a sight impaired individual making it difficult to share accessible resources that patients can actually use. Lastly it was noted there is a lack of unified useful information packs on what social services are available to patients.			
Guerin et al. [25]	Inter-rater agreement: These results indicate that ophthalmologists have low levels of agreement on eligibility criteria. Inter-rater agreement amongst ophthalmologists was poor with κ = 0.16 (95% CI 0.10-0.25) and Krippendorff’s α = 0.30, both indicating weak agreement. The Intraclass Correlation Coefficient (ICC) = 0.31 which further supports poor levels of agreement. When simplified into a binary decision (any impairment compared to none), agreement continues to be low (κ = 0.23; 95% CI 0.14-0.35).	Consultant ophthalmologists showed poor agreement on certification decisions when given identical data, reflecting the lack of standardised visual field criteria. This reliance on subjective judgment leads to unequal access to support services and inaccurate prevalence data on visual impairment. While glaucoma subspecialty training reduced variability, full consensus on eligibility was not achieved. The authors recommend developing unified binocular visual field assessments or automated eligibility algorithms to improve consistency and confidence in CVI decisions.		
	Intra-rater agreement: intra-rater agreement looks to explore how consistent individuals were with their own original decision and interestingly a wide variation (κ = 0.01-0.91; mean < 0.6) was observed which highlights how subjective the matter is.			
	Patterns that emerged from the study: Significant individual variability- some raters certified up to 80% of cases, whereas others certified none. Glaucoma subspecialists showed slightly higher consistency (κ = 0.32) but still poor overall. Variability persisted despite identical data, indicating high subjectivity and lack of standardised criteria. Consultants tended to interpret severe field loss differently due to vague definitions in national guidance.			
Olvera-Barrios et al. [24]	Prevalence and certification gap: Among 8007 patients assessed, 325 (4.3%) met the criteria for visual impairment. However, 84% of these individuals (272 of 325) had not been formally certified as visual impairment when reviewed at the final follow-up visit (mean follow-up duration: 1.87 years).	Over 80% of eligible patients remain uncertified, leading to incomplete data and underestimation of visual impairment prevalence. The presence of an ECLO in consultant-led services did not reduce under-registration, which was influenced by clinician uncertainty, the voluntary nature of the CVI process, and patient reluctance to accept permanent disability. Clinicians’ optimism about visual recovery further contributed. The authors recommend clearer, more objective criteria, structured follow-up pathways, and improved education for both clinicians and patients. Integrating clinical data with CVI records could enhance policy, resource planning, and registration accuracy.		
	Under- registration - Clinician’s views on whether VA is likely to improve or not plays a crucial role in when patients get certified. On many occasions a patient may be eligible for certification but ophthalmologists may opt in to make the decision in their next appointment to see if the vision improves or not. This study identified that 83% remained uncertified even after excluding ≤ 1-year follow-up group (delay in these groups is potentially justifiable as clinician wants to ‘wait and see’ whereas anything beyond 1 year was assumed to be a patient that was lost to under-registration).			

A total of 8,954 patients were included across the reviewed studies. Of these, 947 patients were involved in qualitative or survey-based studies exploring lived experiences and service evaluations [2,19,21], while 8,007 patients with diabetic retinopathy were identified through a hospital-based retrospective cohort study using audit and clinical data [24].

A total of 499 professionals participated across five studies. These included 43 health and social care professionals (e.g., ophthalmologists, nurses, optometrists, ECLOs, and rehabilitation officers) in Boyce et al. [2], 141 multidisciplinary staff across 30 ophthalmology clinics in Llewellyn et al. [20], 129 clinicians (30 consultant ophthalmologists and 99 optometrists) in Bartlett et al. [23], 30 consultant ophthalmologists in Guerin et al. [25], and 148 community-based optometrists in Charlesworth et al. [22].

Most studies were conducted in England, though several also included participants from Scotland, Wales, and Northern Ireland. Sample sizes ranged widely, from 25 to 8,007 participants, reflecting a mix of qualitative interview-based designs and quantitative observational analyses. Participant populations were drawn from hospital eye services, community optometry practices, and local authority or rehabilitation settings, providing diverse insights into the certification and registration processes for visual impairment.

Discussion

This systematic review synthesised qualitative and quantitative evidence on the adult CVI pathway in the UK. When reviewing these eight studies, four recurring themes were identified: limited professional knowledge among professionals regarding CVI registration and its benefits, uncertainty among ophthalmologists in determining eligibility, administrative delays between certification and registration, and the role and value of ECLOs and optometrists. These findings highlight a range of practical issues, most notably persistent knowledge gaps and inconsistent decision-making, which appear to contribute significantly to both delayed and under-registration. This, in turn, will affect epidemiological data, resource allocation, and policy development.

Limited Knowledge Among Professionals Regarding CVI Registration and Its Benefits

Several studies highlighted that many healthcare professionals lack a clear understanding of the CVI process, not just clinically, but in terms of paperwork, referrals, and longer-term support for patients. This contributed to poor communication with patients and resulted in missed opportunities for social and rehabilitative support. For example, clinicians often confused certification, which is a clinical task, with registration, which is a local authority process [2]. This misunderstanding appeared to be widespread and had measurable consequences. In 2016/17, only 87.9% of individuals issued a CVI were subsequently registered, suggesting 12.1% of eligible patients were not captured by the registration process [23]. Patients also expressed mixed awareness of the practical or financial benefits of registration, with some thinking certification had no social benefits [23].

Further evidence emphasised the implications of this knowledge gap on patient care. In their study, 48% of patients received no information about the registration process or its potential benefits. One patient in particular, a female of working age, states, “Looking back, I realise how much advice they didn’t give me” [19] (page 29). In some cases, this lack of communication imposed an emotional burden, contributing to frustration and confusion [19].

Multiple studies also revealed an extensive lack of awareness among both patients and professionals regarding third-sector support services such as those offered by the RNIB, which can be accessed independently of registration [19,21]. Where patients did access such services, they often described them as “invaluable” [2] (page 5), suggesting that improved awareness could improve patient experience even when formal registration is not completed [2].

Implications: we suggest standardisation of cross-professional education on the CVI process, benefits, and eligibility criteria in order to address the barriers highlighted above. Additionally, earlier and consistent referrals to an ECLO, whether or not CVI is required, can benefit patient experience and outcomes.

Uncertainty Among Ophthalmologists in Determining Eligibility

Another commonly occurring theme across literature was inconsistency among ophthalmologists in determining eligibility for CVI certification, highlighting the inherent subjectivity of clinical judgment in this context.

One study reported that agreement among ophthalmologists was 76.0% in cases of SSI and just 51.6% for SI, despite national guidelines being available on the matter [23]. It was also found that LVOs demonstrated similar accuracy to ophthalmologists in these assessments, further highlighting the interpretive nature of certification decisions [23]. This suggests many patients who are eligible for certification may never be certified, subsequently missing out on social support provided in the community.

Another study reinforced that “the whole issue itself is subjective” [2] (page 3) with clinicians reporting that decisions were often influenced by individual interpretation of visual field defects, particularly in cases of fluctuating vision. Technological advances and evolving treatment modalities were noted as additional sources of uncertainty [2]; however, the degree of this impact was not stated. This variability was evident when ophthalmologists were presented with identical clinical data, yet certification decisions differed markedly, with some ophthalmologists certifying around 80% of cases and others certifying almost none [25].

In addition to this issue, some clinicians misunderstood when certification should be offered, often viewing it as something to delay until all treatment options were exhausted, with more than half of the ophthalmologists viewing certification as the “final stage” [2] (page 1). This was particularly an issue when dealing with fluctuating visual acuity in conditions such as AMD, where patients may fluctuate between being certifiable and ineligible based on response to treatment [2]. Another study highlights this, with one of the patients stating, “They said we really should get you registered as visually impaired, but we don’t know what’s going on; we might fix it, and then getting you unregistered is going to be a bit fiddly, so let’s not do it now.” [21] (page 3413).

This misconception contrasts with National Institute for Health and Care Excellence (NICE) Quality Statement 6 on serious eye disorders, which states that healthcare professionals, including ophthalmologists, optometrists, orthoptists, and nurses in secondary care, should ensure that individuals are informed about the benefits of certification and offered a CVI as soon as they are eligible, including while receiving treatment [26]. Ophthalmologists are responsible for formally certifying adults with serious eye disorders as visually impaired, and professionals should provide information about the associated support and services [4]. 

Implications: this variation may stem from unclear guidance or from the difficulty of applying it consistently in complex, real-world settings. Having clearer criteria, backed up by real clinical examples, to make the eligibility process less ambiguous for clinicians could provide significant improvements. Integrating automated prompts into existing electronic health systems, triggered when VA and VF measurements meet predefined thresholds, may help reduce variation. Although there is VA variability, for example, in virtual or injection clinics, VA is not always measured at every visit, meaning some eligible patients might not be identified. Variable testing practices would require alignment before implementation.

Administrative Delays to Registration

Multiple studies identified that delays in administrative processes were a major impediment to timely CVI registration, affecting multiple stages of the pathway. Examples included incomplete documentation, batching of CVIs, and limited administrative support within hospital eye services. Substantial variability in the time taken for patients to move from certification to registration was noted, which ranged from a few weeks to nearly a year [2]. The involvement of ECLOs was described as an important mitigating factor [20]. They relieved consultants of the administrative burden of completing a CVI form, which, in turn, improves clinical workflow [20].

Implications: despite the consistent reports of the administrative inefficiencies, the current literature lacks quantitative data to directly measure their impact on registration outcomes. This shows the need for further evaluation of administrative factors influencing CVI registration. Introducing secure electronic CVI systems that allow real-time sharing with local authorities and automatically track time metrics could help improve communication, minimise delays, and support both local quality improvement and national audits. However, practical issues remain around accessibility: ensuring all clinic users can complete the form within workflows and providing patients with an accessible CVI copy.

The Role and Value of ECLOs and Optometrists

Beyond reducing administrative delays, ECLOs played a wider role in supporting patients’ psychosocial needs and improving the overall efficiency of the CVI process. Their involvement enhanced clinic workflow and patient experience, with studies showing that CVIs were sent to social services within five working days in over 90% of cases where ECLOs were present, reflecting strong adherence to best practice timelines [20]. One participant observed, “You often would find in the olden days piles of CVIs waiting in the consultant’s office - that doesn’t happen anymore” [20] (page 3). ECLO involvement was shown to reduce incomplete CVIs, previously rejected in 10-15% of cases, with both patients and clinicians praising ECLOs for their contribution to the process [2]. In addition to these practical benefits, ECLOs provided essential emotional support and helped patients transition smoothly from clinical care to social and rehabilitative services [20].

However, variation in ECLO training and knowledge was noted. One study reported that only about half of ECLOs had completed the RNIB Eye Clinic Support Studies course, and some were unaware of non-certification support routes such as social prescribing or third-sector referrals [21]. These gaps underscore the need for standardised training and clearer role definitions. It was found that even within consultant-led systems supported by ECLOs, many eligible individuals remained uncertified, reflecting ongoing dependence on both clinician and patient factors [24].

In clinics without ECLOs, optometrists often helped guide patients through the CVI process, but their understanding of CVI, referral of vision impairment (RVI), and low vision leaflet (LVL) pathways was inconsistent, and many were uncertain about referral responsibilities [21]. It should also be noted that, in practice, the LVL pathway was never implemented, and RVI has seen limited use, likely adding to this confusion. It was also observed that clinicians frequently felt uncertain about the process, were unclear about professional roles, and lacked confidence when discussing sight loss [22]. Nonetheless, it was found that LVOs showed a strong ability to assess CVI eligibility [23]. In a cohort of 28 certifiable patients, 88% of optometrists correctly identified eligibility, compared to 75% of ophthalmologists [23]. This supports recent policy changes in Wales, where qualified optometrists can now certify adults with irreversible vision loss in primary care settings, expanding access beyond hospital-based pathways [6].

Differences in practitioner perspectives were also noted. Optometrists and ECLOs tended to consider a patient’s functional status, while ophthalmologists prioritised quantitative measures such as VA [2]. However, it was also suggested that optometrists might adhere more strictly to guidelines and be less focused on patient functionality, leading to more conservative classification [23].

These variations align with CVI guidance, which emphasises that certification categories are advisory and that eligibility should be determined by the consultant ophthalmologist’s professional judgement of how vision loss affects daily function [26]. The guidance also highlights the need to consider each person’s capacity to undertake everyday tasks, their ability to adapt to sight loss, and input from ECLOs or other eye clinic staff to gain a comprehensive understanding of the individual’s circumstances [26].

Implications: given their demonstrated impact on efficiency, patient experience, and coordination, ECLOs and LVOs should be regarded as essential rather than optional components of the CVI pathway. Establishing national standards for training, defining role expectations, and ensuring consistent funding could reduce regional variation and enhance the overall equity and effectiveness of the certification process.

Limitations of This Study

This review is limited by the small number of eligible studies, many of which were qualitative and conducted within single centres or specific regions. However, the inclusion of both qualitative studies and a large retrospective cohort provides complementary perspectives that help contextualise these findings. Even so, the findings may not fully reflect experiences across the wider UK. Differences in local service structures and resources could mean that challenges vary between areas. Qualitative studies may also over-represent the perspectives of individuals who are particularly engaged or dissatisfied with the CVI process, potentially introducing response bias into the portrayal of patient and professional experiences. Reporting bias was not formally assessed, and Grading of Recommendations Assessment, Development and Evaluation (GRADE) was not applied, as the predominantly qualitative, mixed-methods evidence and narrative synthesis precluded formal certainty appraisal. Nonetheless, the possibility of publication or selective reporting bias cannot be excluded given the limited and heterogeneous evidence base. Future national data collection would help build a clearer, more representative picture of how the CVI process functions across the country.

## Conclusions

Despite clear national guidance, the UK CVI pathway remains poorly understood and inconsistently applied. Across studies, substantial numbers of eligible adults are neither certified nor registered, which results in limited access to social and rehabilitative support and weakened epidemiological data used for public-health planning. While implementation of system-level change depends on local funding, workforce capacity, and service configuration, several other priorities emerge consistently across the evidence. Addressing professional knowledge gaps about certification, registration and associated benefits; reducing uncertainty in eligibility decisions through clearer, functionally orientated guidance; and tackling administrative delays (including through accessible electronic CVI systems) are key priorities. ECLOs and trained community optometrists clearly have a valuable role to play in the CVI process. At present, their role appears to be an optional part of the process, but recognising them as an essential role in CVI registration and formally embedding their roles in the national CVI framework could improve the speed and fairness of access to support and is likely to offer efficiency gains, suggesting a potentially cost-effective use of resources. National standards for training, role clarity, and data reporting would also help reduce the current postcode lottery in patient outcomes.

## Appendices

Search strings

PubMed:

("Vision Disorders"[Mesh] OR "Blindness"[Mesh] OR "Vision, Low"[Mesh] 

OR "Visual Impairment"[tiab] OR "sight impairment"[tiab] 

OR "severely sight impaired"[tiab] OR "severe sight impairment"[tiab] 

OR "low vision"[tiab] OR "visual disability"[tiab])

AND

("Certification"[tiab] OR "Certificate of Vision Impairment"[tiab] 

OR "CVI form"[tiab] OR "CVI process"[tiab] OR "CVI certificate"[tiab] 

OR "registration"[tiab] OR "registering"[tiab] 

OR "vision impairment registration"[tiab] 

OR "sight impaired registration"[tiab] 

OR "local authority registration"[tiab] 

OR "visual impairment registration"[tiab])

AND

("Health Services Administration"[Mesh] OR "Patient Registration"[Mesh] 

OR "Referral and Consultation"[Mesh] OR "Health Services Accessibility"[Mesh] 

OR "Health Services Needs and Demand"[Mesh]

OR "administration"[tiab] OR "service delivery"[tiab] 

OR "process"[tiab] OR "pathway"[tiab] OR "workflow"[tiab]

OR "barrier*"[tiab] OR "facilitator*"[tiab] 

OR "delay"[tiab] OR "delays"[tiab] OR "under-certification"[tiab] 

OR "non-certification"[tiab] OR "incomplete certification"[tiab])

AND

("Ophthalmology"[Mesh] OR "Ophthalmologists"[Mesh] OR "Optometry"[Mesh] 

OR "Optometrists"[Mesh] OR "Allied Health Personnel"[Mesh]

OR "Eye Clinic Liaison Officer"[tiab] OR ECLO[tiab] 

OR "ophthalmologist*"[tiab] OR "optometrist*"[tiab]

OR "eye clinic"[tiab] OR "hospital eye service"[tiab])

AND

("United Kingdom"[Mesh] OR "UK"[tiab] OR "United Kingdom"[tiab] 

OR England[tiab] OR Scotland[tiab] OR Wales[tiab] OR "Northern Ireland"[tiab]) 

Medline: 

1. exp Vision Disorders/ OR exp Blindness/ OR exp Vision, Low/

2. (visual impairment or sight impairment or low vision or visual disability or severely sight impaired or severe sight impairment).ti,ab.

3. 1 OR 2

4. (Certificate of Vision Impairment or CVI form or CVI process or CVI certificate or registration or vision impairment registration or sight impaired registration or visual impairment registration or local authority registration).ti,ab.

5. 4

6. exp Health Services Administration/ OR exp Patient Registration/ 

OR exp Referral and Consultation/ OR exp Health Services Accessibility/

OR exp Health Services Needs and Demand/

7. (administration or service delivery or process or pathway or workflow 

or barrier* or facilitator* or delay or delays or under-certification 

or non-certification or incomplete certification).ti,ab.

8. 6 OR 7

9. exp Ophthalmology/ OR exp Ophthalmologists/ OR exp Optometry/ 

OR exp Optometrists/ OR exp Allied Health Personnel/

10. (ophthalmologist* or optometrist* or ECLO or 

Eye Clinic Liaison Officer or eye clinic or hospital eye service).ti,ab.

11. 9 OR 10

12. exp United Kingdom/ OR (UK or United Kingdom or England or Scotland 

or Wales or Northern Ireland).ti,ab.

13. 3 AND 5 AND 8 AND 11 AND 12

**Table 6:** Summary of benefits and concessions available to individuals certified and/or registered as Sight Impaired or Severely Sight Impaired in the UK. The table outlines national and local entitlements dependent on certification or registration status, as well as benefits that may be available based on medical, physical, or financial need. A tick (✔) indicates eligibility; a cross (✘) indicates ineligibility. Information for this table was provided directly from the Royal National Institute of Blind People.

Entitled to – dependent on certification or registration	Sight Impaired	Severely Sight Impaired
Blind person’s tax allowance	✘	✔
Television license fee reduction	✘	✔
Blue Badge Scheme – car parking	✘	✔
Free postage – “Articles for the blind”	✔	✔
Free National Health Service (NHS) eye examination	✔	✔
Disabled Persons Railcard and travel assistance + rail concession if travelling with a companion without a railcard	✔	✔
Free bus travel	✔	✔
Free directory enquiries	✔	✔
Cinema pass for carer	✘	✔
Protection under the equality act	✔	✔
Assessment by social services	✔	✔
Free permanent loan of radios, audio players and television sound receivers (check with local authority if available)	✘	✔
Value-added tax exemption on products designed or adapted for your own personal or domestic use	✔	✔
May be entitled to - dependent on level of medical/physical need	Sight Impaired	Severely Sight Impaired
Personal Independence Payment (PIP) (adult disability payment in Scotland)	✔	✔
Attendance allowance	✔	✔
Pension age disability payment	✔	✔
Employment and support allowance	✔	✔
Council tax disability reduction	✔	✔
Free ticket for a guide at theatres, galleries or tourist attractions	✔	✔
Local travel schemes and concessions - for example taxi card, carers pass (subject to local variations)	✔	✔
Vehicle tax exemption - if in receipt of enhanced mobility PIP or higher rate mobility disability living allowance	✔	✔
Free NHS prescriptions via FP92A form*	✔	✔
May be entitled to - means tested	Sight Impaired	Severely Sight Impaired
Carer’s Allowance	✔	✔
Carers Support Payment	✔	✔
Tax Credits	✔	✔
Housing Benefit	✔	✔
Universal Credit	✔	✔
Pension Credit	✔	✔
Exemption from “non-dependants” deduction from universal credit, pension credit, housing benefit and council tax benefit	✔	✔
Help towards residential or nursing home fees	✔	✔
Help with telephone installation charges and line rental	✘	✔
Free NHS Prescriptions via NC1(exemption due to receipt of a qualifying benefit)	✔	✔

**Table 7:** Glossary of key terms and abbreviations used in the review.

Term	Definition
Sight Impaired (SI)	Individuals with significantly reduced vision who still have some useful vision [27].
Severely Sight Impaired (SSI)	Individuals with very severe visual impairment who are unable to read standard print even with optimal correction using glasses or contact lenses [27].
Visual Acuity (VA)	Describes sharpness of an individual’s vision at a distance, used as a metric measure of assessing vision [28].
Visual Fields (VF)	Defined as the extent an area is visible to an individual when they fixate their vision on one point [29].
Local Social Services Department (LSSD)	Services provided by a city’s council to help with day to day activities of an individual who requires more support due to a pre-existing medical or mental health condition, disability, or complicated living situations [30].
Eye Care Liaison Officer (ECLO)	Individuals with a nationally recognised qualification in Eye Clinic Support Studies who work within eye care services to provide emotional, practical, and informational support to people with visual impairment, and to help link patients and their families with relevant health, social care, and voluntary sector services [31].
Low Vision Optometrist (LVO)	Optometrists trained in low vision care who assess visual function and provide practical interventions to maximise functional vision in people with visual impairment [32].
